# Immunogenicity of RSV F DNA Vaccine in BALB/c Mice

**DOI:** 10.1155/2016/7971847

**Published:** 2016-09-05

**Authors:** Erdal Eroglu, Ankur Singh, Swapnil Bawage, Pooja M. Tiwari, Komal Vig, Shreekumar R. Pillai, Vida A. Dennis, Shree R. Singh

**Affiliations:** ^1^Center for NanoBiotechnology Research, Alabama State University, Montgomery, AL, USA; ^2^Faculty of Engineering, Bioengineering Department, Celal Bayar University, Muradiye, Manisa, Turkey; ^3^College of Medicine, University of South Alabama, Mobile, AL, USA; ^4^Yerkes National Primate Research Center, Emory University, Atlanta, GA, USA

## Abstract

Respiratory syncytial virus (RSV) causes severe acute lower respiratory tract disease leading to numerous hospitalizations and deaths among the infant and elderly populations worldwide. There is no vaccine or a less effective drug available against RSV infections. Natural RSV infection stimulates the Th1 immune response and activates the production of neutralizing antibodies, while earlier vaccine trials that used UV-inactivated RSV exacerbated the disease due to the activation of the allergic Th2 response. With a focus on Th1 immunity, we developed a DNA vaccine containing the native RSV fusion (RSV F) protein and studied its immune response in BALB/c mice. High levels of RSV specific antibodies were induced during subsequent immunizations. The serum antibodies were able to neutralize RSV* in vitro*. The RSV inhibition by sera was also shown by immunofluorescence analyses. Antibody response of the RSV F DNA vaccine showed a strong Th1 response. Also, sera from RSV F immunized and RSV infected mice reduced the RSV infection by 50% and 80%, respectively. Our data evidently showed that the RSV F DNA vaccine activated the Th1 biased immune response and led to the production of neutralizing antibodies, which is the desired immune response required for protection from RSV infections.

## 1. Introduction

Respiratory syncytial virus (RSV), a member of genus* Pneumovirus* and classified in the family Paramyxoviridae, is the most common cause of severe disease of the lower respiratory tract in infants and the elderly especially in developing countries [1, 2]. There are also some reports claiming that RSV could lead to severe repeated infections such as recurrent wheezing, pneumonia, or asthma in later childhood [3]. Worldwide, the number of RSV-associated cases is estimated to be 33 million and the number of deaths up to 234,000 in children younger than 5 years old in spite of the fact that those numbers are lower in the USA due to the precautions against RSV [4, 5]. Besides the young children, the hospitalization rate of elderly people above 50 years old may be the same as influenza cases [2]. RSV vaccine development efforts such as inactivated RSV, live-attenuated RSV, or subunit vaccines are underway. However, despite over five decades of intensive research on developing a RSV vaccine, there is no approved vaccine or drug available [6]. Instead of vaccine, some researchers have been attempting to develop prophylactic antibody therapies targeting RSV F protein [7, 8]. Antiviral drugs such as ribavirin (a nucleoside analog), which targets hepatitis C and other viruses including RSV, ALS-8176 (a new nucleoside analog), and GS5806 (pyrazolo[1,5-a]pyrimidine based RSV fusion inhibitor), and neutralizing monoclonal antibodies such as Palivizumab (Synagis*™*) and Motavizumab (Numax), are administered to infants at high risk of developing respiratory diseases [9–12]. As an alternative to expensive therapies, a vaccine conferring long lasting immunity is a less expensive and more efficient option against recurrent RSV infections [10]. Due to frequent antigenic variations of RNA viruses (RSV, influenza virus, and rhinovirus), developing a vaccine with complete protection is challenging. The incomplete immunity in response to natural RSV infections is responsible for repeated infections. RSV vaccine studies in the 1960s using formalin inactivated RSV (FI-RSV) consisting of the whole virus exacerbated the disease and even in some cases resulted in deaths because of the elevated T helper type-2 (Th2) mediated immune response [1, 13]. In addition, using a vector expressing RSV antigens is found far safer than subunit or inactivated RSV immunization [14]. With these important immunological responses, a safe and stable vaccine with long lasting immunity is an urgent need for the public.

The outer surface glycoproteins, fusion (F) and attachment (G), of RSV are known antigenic proteins that induce the humoral and cellular immune responses and are targets of antigen presenting cells [15]. The RSV F protein mediates the fusion of the virus particle into the host by merging the virion envelope with the host cell membrane following virion attachment using the G protein. In addition, the F protein facilitates fusion of neighboring normal cells with infected cells, thus creating multinuclear giant cells called syncytia, which characterizes RSV infection [9, 16]. The RSV F protein is highly conserved among the different RSV strains compared to other RSV proteins [16]. On the other hand, the variability of the G amino acid sequence among various RSV strains is high [17]. Furthermore, previous reports demonstrated that RSV F vaccines provide protection against both RSV A and RSV B strains by producing neutralizing antibodies [8, 14, 18, 19], whereas RSV G vaccines prominently induced a Th2 biased immune response, thereby enhancing the severity of the disease in subsequent RSV infections [20, 21].

The helper T lymphocytes activate either B cells, which produce specific antibodies, or cytotoxic T lymphocytes, which are responsible for the clearance of RSV infected cells. Cell-mediated protective immunity is important in the clearance of infected cells. However, cell-mediated immunity on its own is not sufficient to provide complete protection against pathogens. Therefore, it is necessary to have memory B cells activating neutralizing antibodies upon reinfection. Although DNA vaccines are not highly immunogenic as compared to whole pathogen or protein vaccines, DNA vaccines have the advantage of expressing the native form of the antigen produced* in vivo* and inducing strong T and B cells responses. The changes in the epitope regions of the antigen may shift the immune response leading to unwanted allergic immune reactions as seen in the FI-RSV vaccine trials [6, 22, 23]. A highly immunogenic RSV F protein with conserved sequence would be a desirable DNA vaccine candidate for protection from repeated RSV infections. Our group has previously developed a DNA vaccine containing immunogenic regions of RSV F protein (residues 412–524) and showed that the DNA vaccine provides partial protection in BALB/c mice when combined with cholera toxin (CTA_2_B) adjuvant [24].

In this study, we developed a full-length RSV F DNA vaccine that was able to induce predominantly a Th1 type response without using any adjuvant. The antibody response in serum was significantly enhanced with subsequent immunizations. The sera from immunized animals were able to neutralize RSV* in vitro*. The protection afforded by the DNA vaccine was not complete and thus necessitates design and development of other methods of vaccines. A combinatorial concept that can take advantages of various vaccines such as a prime vaccine (DNA vaccine) followed with booster vaccines (subunit or recombinant protein vaccines) may lead to complete protection from RSV.

## 2. Materials and Methods

### 2.1. Materials

Restriction enzymes (RE)* Not*I and* Bam*HI, T4 DNA ligase, Eagle's minimal essential medium (MEM), Hank's balanced salt solution (HBSS), fetal bovine serum (FBS), L-glutamine (100 mM), antibiotics, TrypLE*™*, 7-aminoactinomycin D (7-AAD), Nucleofector*™* electroporation kit for Cos-7 cells, TaqMan master mix 2x, real time probe, primers, superscript II reverse transcriptase, and RNase later solution were all obtained from Life Technologies*™* (Carlsbad, CA, USA). All DNA and RNA isolation kits were purchased from QIAGEN*™* (Valencia, CA, USA). MEM was supplemented with 10% FBS (MEM-10), penicillin (45 *μ*g/mL), streptomycin (100 *μ*g/mL), kanamycin (75 *μ*g/mL), and L-glutamine (1 mM). Human epithelial type 2 (HEp-2) and monkey kidney (Vero and Cos-7) cells were obtained from American Type Culture Collection (ATCCTM, Manassas, VA, USA).

### 2.2. Animals and RSV Stock Preparation

BALB/c female mice (4–6 weeks old) were purchased from Charles River Laboratories (Wilmington, MA). The animals were housed under standard approved conditions with a cycle of 12 h of light and 12 h of darkness and provided daily with sterile food and water* ad libitum*. For all immunization studies, an approved protocol by the Alabama State University Institutional Animal Care and Use Committee was followed. Human RSV long strain was purchased from the American Type Culture Collection (ATCC, Manassas, VA, ATCC # VR-26) and propagated in HEp-2 cells (ATCC # CCL-23). HEp-2 cells were grown in tissue culture flasks in MEM supplemented with 10% FBS and antibiotics. Human RSV was added to the cell monolayer, and virus adsorption was carried out for 1 h at 37°C in a humidified atmosphere with 5% CO_2_. MEM with 2% FBS was added to the flask and infection of cells was observed for an additional 3-4 days. RSV infected cells were centrifuged at 3,000 ×g at 4°C to remove cellular debris, aliquoted, and stored at −80°C until they were used.

### 2.3. Construction of Recombinant RSV F DNA Vaccine

**Figure 1:**
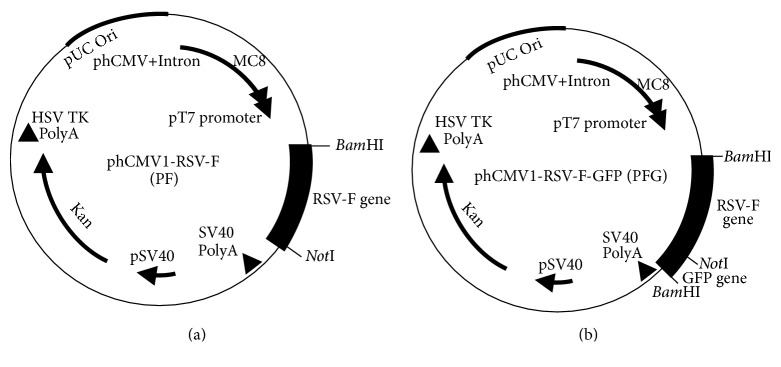
Construction of RSV F gene into phCMV1 vector. (a) RSV F gene sequence was amplified with PCR reaction and cloned into* Bam*HI and* Not*I RE sites on phCMV1 vector. (b) GFP tag was amplified with PCR reaction and inserted into* Not*I RE sites at the 3′ end of the RSV F gene.

The RSV F DNA sequence originally published by Collins et al. [25] was full-length RSV F gene synthesized by Epoch labs (Missouri City, TX, USA) and amplified by polymerase chain reaction (PCR) using forward and reverse primers shown in Table 1. Both the RSV F gene and the phCMV1 DNA vector were digested with* Bam*HI and* Not*I RE enzymes. The purified DNA pieces (using QIAGEN gel extraction kit) were ligated using the T4 DNA ligase enzyme and transformed into competent cells of* Escherichia coli* DH5*α*. For selection, competent cells were grown on kanamycin supplemented Luria Bertani (LB) agar. Clones containing the RSV F gene in the phCMV1 vector were named PF (Figure 1(a)).

### 2.4. Construction of phCMV1 Vector Containing GFP Gene

The GFP gene was amplified by PCR using previously published GFP plasmid vector [26] as a template, forward and reverse primers (Table 1) with* Bam*HI and* Not*I restriction sites, respectively. The amplified GFP gene was inserted into the phCMV1 vector following the same protocol and conditions as described above. Clones containing the GFP gene in the phCMV1 vector were named PG. The third vector, containing RSV F and GFP, was also cloned and named PFG (Figure 1(b)). All vectors were purified using the QIAGEN Endofree Giga kit and the purified DNA vector aliquots (100 *μ*g/100 *μ*L) were stored at −80°C until used.

### 2.5. *In Vitro* Transfection and Expression of RSV F Protein

Nucleofector*™* (Lonza, Germany) electroporation protocol was used for* in vitro* gene transfection following the manufacturer's instructions in Cos-7 cells using the Amaxa*™* Nucleofector II electroporation device (Lonza, Germany). The GFP labeled RSV F gene construct was used for immunofluorescence imaging and flow cytometry, whereas the RSV F gene construct was used in RT-PCR analysis. Transfected cells with RSV F DNA were incubated for 3 days at 37°C to allow for protein expression* in vitro*. After the incubation time, images showing protein expression with green color were taken using an immunofluorescence microscope. Also, the cells were used for flow cytometry to detect the green fluorescence of GFP labeled RSV F protein. Transfection and expression protocol was followed as described above for RSV F DNA to detect the RSV F mRNA by RT-PCR.

### 2.6. Quantitative PCR (qPCR) Analysis for Detection of RSV F Gene

In order to analyze the transfection efficiency, Cos-7 cells were transfected with the PF construct using the Nucleofector electroporation gene transfection following manufacturer's instructions. Total RNA was isolated from harvested cells and 1 *μ*g of RNA was converted into cDNA using the superscript reverse transcriptase enzyme following manufacturer's protocols. RSV F mRNA specific primers, probe, and experimental protocol for qPCR were adapted from Mentel et al. [27]. The qPCR reaction was carried out with reverse and forward primers (Table 1) using Applied Biosystems ViiA 7 real time PCR (Applied Biosystems International, Foster City, CA, USA). Each qPCR reaction was run in duplicate along with water as a negative control. Dilutions of the RSV F gene amplicon (10^0^–10^8^ copy numbers) were used to prepare the standard curve. Each experiment was repeated twice from the transfection step in duplicate.

### 2.7. Immunization of BALB/c Mice and Determination of Antibody Response

Animal studies were performed according to the National Institute of Health (NIH, Bethesda, MD) guidelines following a protocol approved by the Alabama State University Institutional Animal Care and Use Committee. Animals were housed under standard approved conditions and provided daily with sterile food and water* ad libitum*. Six-to-eight-week-old female BALB/c mice (Charles River Laboratories Inc., Wilmington, Massachusetts) were immunized intramuscularly (i.m.) with PBS (300 *μ*L) and PF DNA (50 *μ*g/300 *μ*L in PBS) to each thigh muscle on days 1, 15, and 29. The RSV control group was immunized intranasally with 2 × 10^5^ plaque forming units (pfu) of live RSV long strain (200 *μ*L) twice on day 1 and day 2 by slow application to the nasal nares. Serum was collected via retro-orbital bleeding from all groups of mice on days 0, 14, 28, and 49 to determine the RSV specific antibody response. Saliva was collected by injecting carbachol (0.25 *μ*g/mouse) intraperitoneally on the same days as sera collections. Serum and saliva samples were stored at −80°C until analysis.

Sera and saliva samples collected from the mice were analyzed for antibody response and isotyping. To analyze the anti-RSV F-specific antibody response, ELISA plates were coated with UV-inactivated RSV (10^4^ pfu/well) in 100 *μ*L of carbonate buffer (pH 9.2) and incubated overnight at 4°C in a humidified atmosphere. Plates were blocked with 3% milk powder and then incubated with 100 *μ*L of samples at room temperature for 1 hour. Goat anti-mouse HRP-conjugated secondary antibody (100 *μ*L of 1 : 2,000 dilution) specific to isotypes IgA, IgG1, IgG2a, and IgG2b was added to the ELISA plates and incubated at room temperature for 1 hour. The ELISA plates were washed and the enzymatic reaction was developed and absorbance was read at 450 nm using a Tecan ELISA reader (Tecan, Research Triangle Park, NC, USA).

### 2.8. Viral Neutralization Assay

The viral neutralization assays for the mice sera samples were performed according to the protocols of Singh et al. with slight modifications [18]. Briefly, heat inactivated sera (56°C for 30 min; 25 *μ*L and 12.5 *μ*L per well) from all groups of mice (PBS, RSV, and RSV F DNA) were mixed with 1 × 10^3^ pfu of RSV and incubated at 4°C for 2 hours. Approximately, 1.5 × 10^3^ HEp-2 cells were mixed with sera+RSV mixture in a 96-well plate followed by incubation at 37°C in a CO_2_ incubator for 3 days. HEp-2 cells alone and HEp-2 cells infected with RSV (1 × 10^3^ pfu) were used as negative and positive controls, respectively. Cells were washed with 1x PBS (pH 7.0) before fixing the cells by incubating in 80% acetone (v/v) at 4°C for 15 minutes. For the ELISA assay, the plate was blocked with 3% milk and incubated with the primary antibody, goat anti-RSV (1 : 500 dilution), and then the secondary antibody, rabbit anti-goat IgG-HRP (1 : 2000 dilution) at room temperature for 1 hour. The plate was washed and the enzymatic reaction was developed with the TMB substrate (KPL, Gaithersburg, Maryland, USA) followed by reading the absorbance at 450 nm in the ELISA reader (TECAN, US Inc., Durham, NC, USA). The same protocol was followed for the immunofluorescence microscopy analysis except for the secondary antibody, rabbit anti-goat IgG-FITC (1 : 2000 dilution). The cell nuclei were stained with antifade-DAPI, and merged images were taken using the FITC and DAPI channel in the immunofluorescence microscope (Nikon Eclipse Ti, Nikon Instruments Inc., Melville, USA).

### 2.9. Statistical Analysis

qPCR, ELISA, and virus neutralization assay data are presented as means and standard deviations; statistical analysis of the data was performed using Sigma plot version 11.0 (Systat Software, Inc., Germany). Differences between the means of the four experimental groups were determined using one-way analysis of variance (ANOVA) Tukey's test with the significance level of 1%.

## 3. Results

### 3.1. Expression of DNA Vaccine* In Vitro*

**Figure 2:**
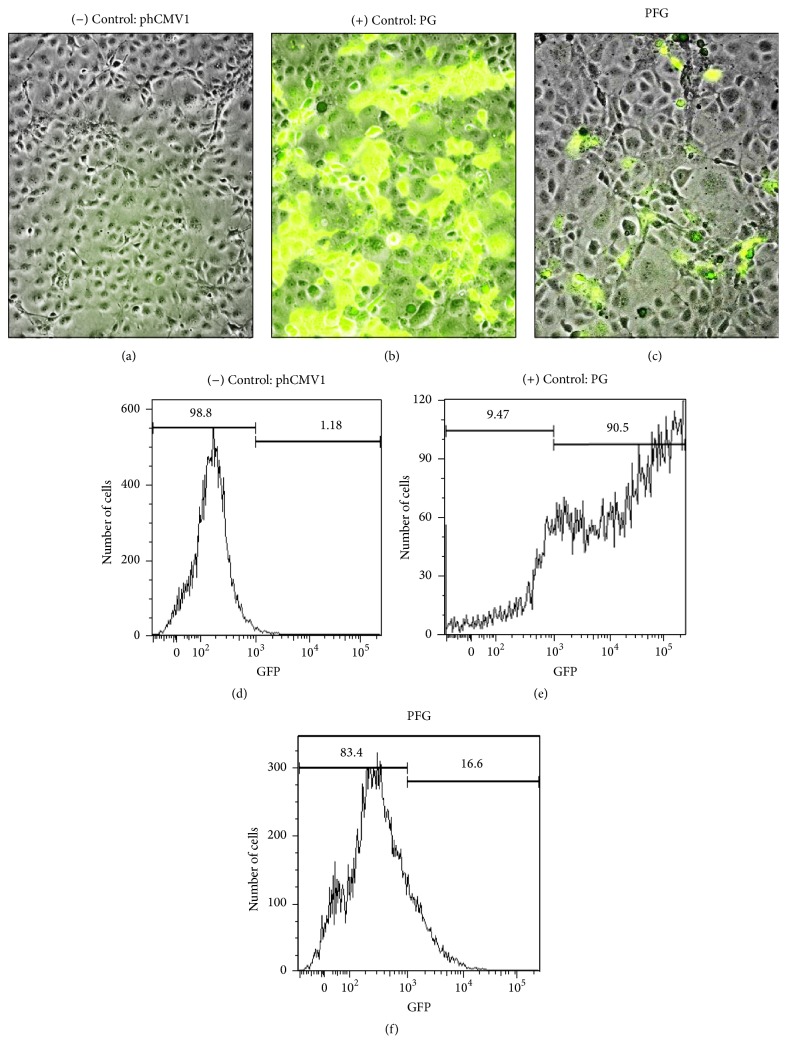
Expression of RSV F protein in Cos-7 cells. Visual and quantitative analyses demonstrated that RSV F protein was expressed* in vitro*. Immunofluorescence microscopy of (a) phCMV1 (negative control), (b) phCMV1+GFP (positive control), and (c) PFG (DNA vaccine) transfected cells. Flow cytometric analysis of transfected cells: (d) Cos-7 cells (negative control), (e) phCMV1+GFP (positive control), and (f) PFG (DNA vaccine).

The RSV F gene was cloned into the phCMV1 vector between the* Bam*HI and* Not*I restriction enzymes sites. Positive clones were verified by RE digestion (*Bam*HI and* Not*I) and DNA sequencing. Recombinant clones containing the RSV F gene in the phCMV1 vector were named PF. To test the expression efficiency of the RSV F protein expression* in vitro*, the RSV F gene containing the GFP gene was similarly cloned into the phCMV1 vector generating the PFG clone. The PF and PFG clones were used to transfect Cos-7 cells. Three days after transfection, green fluorescence signals were analyzed (Figures 2(a)–2(c)). The PG clone showed a high level of GFP protein expression (Figure 2(b)). The PFG vector expressed RSV F and GFP proteins (Figure 2(c)) although at a much lower level compared to the PG clones. Transfected cells were trypsinized and protein expression was detected in flow cytometry (Figures 2(d)–2(f)). The transfection efficiency of the PG clone (phCMV1-GFP) was over 90% (Figure 2(e)) while the efficiency of the PFG clone (RSV F-GFP) was over 16% (Figure 2(f)) compared to the negative (phCMV1) clone.

**Figure 3:**
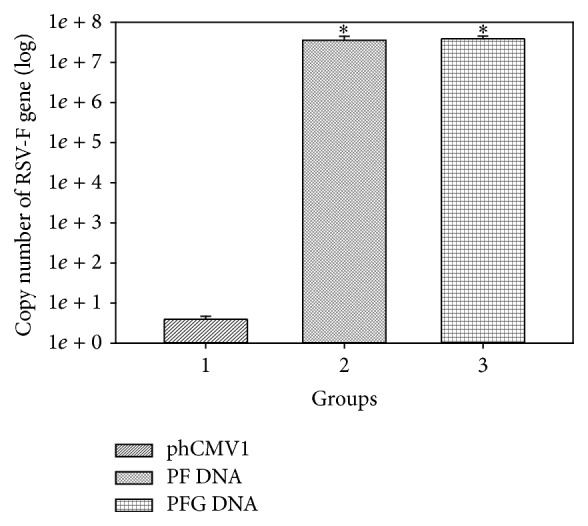
qPCR data showing the transcription of RSV F gene in PF/PFG transfected Cos-7 cells. Transcription of RSV F mRNA was >10^7^-fold higher than mock transfected cells (negative control, <10^1^). ^*∗*^Significantly different (*P* < 0.01).

In addition to immunofluorescence and flow cytometry analyses, the transcription efficiencies of the PF and PFG clones were quantified by qPCR analysis in Cos-7 cells using RSV F specific primers (Figure 3). The RSV F mRNA copy numbers for both clones (PF and PFG) were significantly higher (4 × 10^7^) compared to the RSV F mRNA copy number for the negative control (mock transfected cells, <10^1^). Thus, we confirmed, using three different methods, that PF clones were expressing RSV F protein* in vitro*.

### 3.2. Analysis of RSV Specific Antibody Response

**Figure 4:**
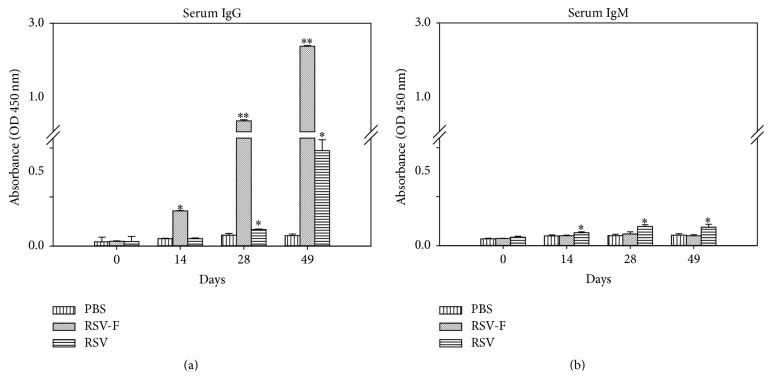
(a) IgG antibody response and (b) IgM antibody response against RSV specific antigens. Serum samples (PBS, RSV infected, and PF DNA-immunized mice) were collected from BALB/c mice on days 0, 14, 28, and 49 and IgG antibody responses were detected by ELISA. Data is presented as an average of triplicates performed twice; error bars represent standard deviations. ^*∗*^Significantly different (*P* < 0.01) from PBS group; ^*∗∗*^significantly different (*P* < 0.01) from PBS and PF DNA groups. *P* values (*P* < 0.01) were calculated using ANOVA, Tukey's test.

The humoral immune response induced by immunizing mice with PBS, the RSV F DNA vaccine, or RSV was determined by measuring RSV specific serum and saliva antibody titers using ELISA. Serum and saliva samples were collected from BALB/c mice at 2-week intervals following each immunization. Animals vaccinated with RSV F DNA and RSV showed significantly higher (*P* < 0.01) amount of serum IgG levels compared to the PBS negative control group (Figure 4(a)). Saliva samples from same groups showed no significant RSV specific IgG antibody response except for saliva samples from RSV vaccinated mice collected on day 49 (data not shown). RSV specific IgM antibody was detected only in serum samples (not in saliva) from RSV F immunized mice during all immunization periods (Figure 4(b)). IgM, a basic immunoglobulin produced in B cells, is the first antibody produced in response to an initial exposure to an antigen [28].

### 3.3. Isotyping of RSV Specific IgG Antibody

**Figure 5:**
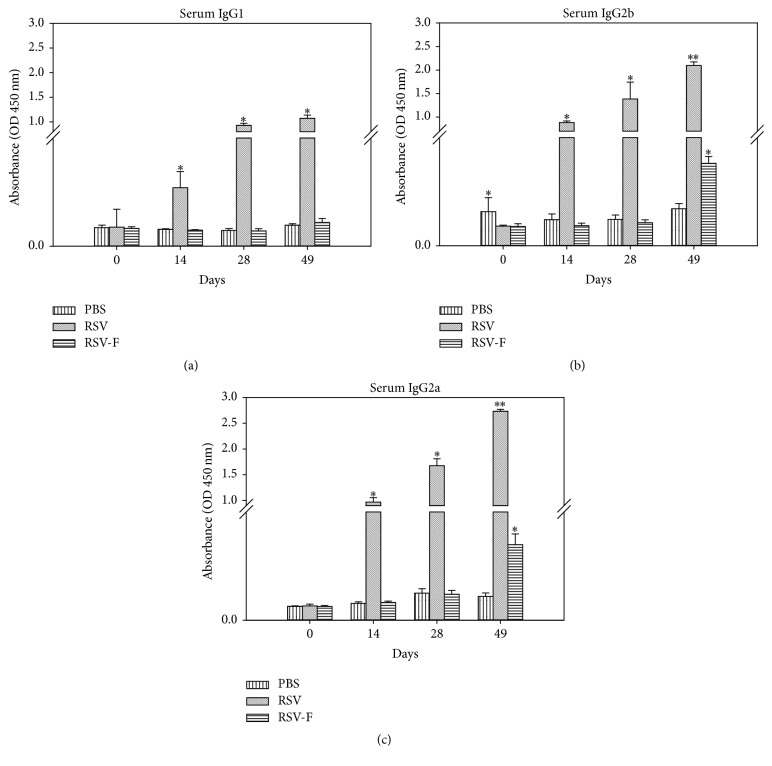
IgG isotypes; (a) IgG1, (b) IgG2b, and (c) IgG2a antibody response against RSV specific antigens. Serum samples (PBS, RSV infected, and PF DNA-immunized mice) were collected from BALB/c mice on days 0, 14, 28, and 49 and IgG isotypes were detected by ELISA. Data is presented as an average of triplicates performed twice; error bars represent standard deviations. ^*∗*^Significantly different (*P* < 0.01) from PBS group; ^*∗∗*^significantly different (*P* < 0.01) from PBS and PF DNA groups. *P* values (*P* < 0.01) were calculated using ANOVA, Tukey's test.

Since the Th1 immune response is important in providing protective immunity against RSV infection, we also analyzed and compared the Th1 (IgG2a) and Th2 (IgG1, IgG2b) specific immune responses. Antibody isotyping of serum samples showed significant levels of IgG1 (Figure 5(a)), IgG2b (Figure 5(b)), and IgG2a (Figure 5(c)) antibodies after day 14 of immunization and levels continued to increase on day 28 and day 49 in RSV infected mice. In the RSV F DNA immunized mice, IgG2b and IgG2a production was stimulated only at day 49 while no IgG1 production was detected in all serum samples. The IgG2a isotype antibody response, specific for the Th1 mediated response, was significantly higher than the IgG1 and IgG2b at all immunizations. All IgG1/IgG2a and IgG1/IgG2b ratios were calculated (Table 2) and all of the ratios were constantly lower than 1 clearly demonstrating a Th1-biased response following either RSV infection or RSV F vaccination. A Th1 (IgG1/IgG2a < 1 and IgG2b/IgG2a < 1) response was prominent in both RSV infected and RSV F DNA immunized mice at day 49, the time when the antibody level was highest.

### 3.4. RSV F DNA Vaccine Stimulates RSV Specific Neutralizing Antibodies

**Figure 6:**
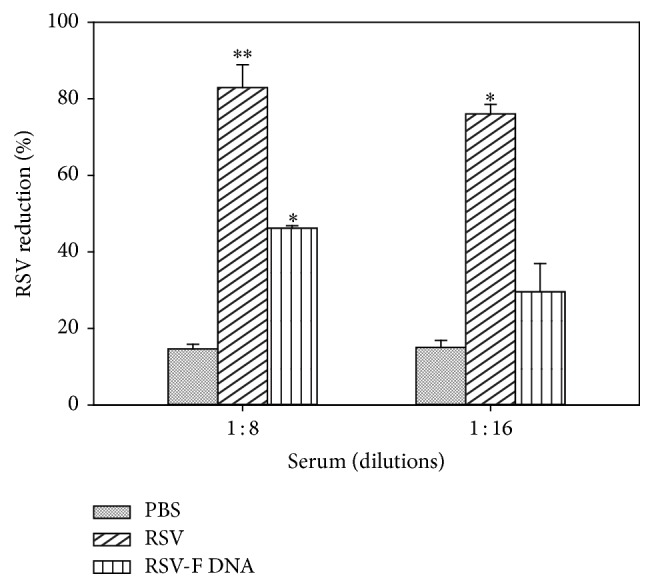
Neutralization of RSV on Hep-2 cells, ELISA. The number of RSV mixed with different dilutions of serum (1 : 16 and 1 : 8) from mice groups (PBS, RSV, infected and PF DNA-administrated mice) reduced significantly. ELISA was used to detect RSV reduction. Two sera pools from each group of mice were run in duplicate. Bar graphs are represented as means with standard deviations. ^*∗*^Significantly different (*P* < 0.01) from PBS group; ^*∗∗*^significantly different (*P* < 0.01) from PBS and PF DNA groups. *P* values (*P* < 0.01) were calculated using ANOVA, Tukey's test.

**Figure 7:**
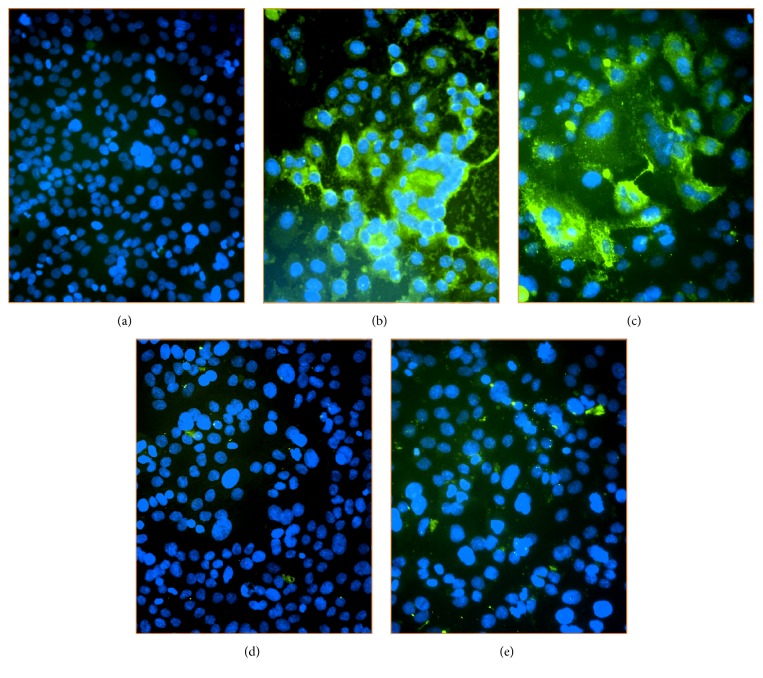
Neutralization of RSV on Hep-2 cells, immunofluorescence microscopy. (a) Negative control: uninfected Hep-2 cells, (b) positive control: RSV infected cells only, (c) PBS group: RSV mixed with sera collected from PBS injected mice, (d) RSV sera: RSV mixed with sera collected from RSV infected mice, and (e) PF DNA sera: RSV mixed with sera collected from PF-immunized mice.

We also tested the efficacy of serum antibodies in neutralizing RSV infection* in vitro* using ELISA. ELISA data indicated that RSV specific neutralizing antibodies from RSV F DNA immunized mice serum reduced the infectivity of RSV by 46% and 30% in 1 : 8 serum dilution and 1 : 16 serum dilution, respectively (Figure 6). Consistently, serum from RSV infected mice showed higher RSV reduction with 82% and 76% in 1 : 8 serum dilution and 1 : 16 serum dilution, respectively. The data of the neutralization assay was in accordance with the antibody response data. Also, the ELISA data for RSV neutralization was confirmed with an immunofluorescence assay. The same experiment was repeated under the same conditions and reduction of RSV infection was visualized by a decrease in the FITC signal in immunofluorescence microscopy (Figure 7). RSV infection was visibly observed in the HEp-2 cells incubated with serum from PBS mice compared to untreated Hep-2 cells (Figures 7(a) and 7(b)). The intensity of the FITC signal of the PBS group was detected as strong as the positive control (without serum), whereas no green signal was observed on the cells incubated with RSV+serum from RSV infected mice (Figures 7(c) and 7(d)). On the other hand, RSV immunized serum considerably neutralized RSV infection in HEp-2 cells and insignificant green signals were detected (Figure 7(e)). Immunofluorescence microscopy observations confirmed the results of the ELISA neutralization assay and the antibody response data.

## 4. Discussion

As with other pathogenic infections, RSV initially activates the innate response and subsequently develops cellular and humoral immunity. The cellular immunity is needed to clear the infection, whereas the humoral immune response (antibody mediated) is required for protection from initial and subsequent RSV infections. During the 1960s, vaccinations performed with FI-RSV suggested that FI-RSV immunization leads to a predominant Th2 type allergic response. Whereas wild type RSV activates T helper type 1 (Th1) skewed immune providing protection against RSV disease [1]. Thus, the Th1 type immune response is desired for protection against natural RSV infections. To understand the mechanism and type of immune responses for FI-RSV immunizations, different animal models such as monkeys [29], bovine [30], mice [31], and cotton rats [23, 32] were tested. All models challenged with wt RSV following the immunization with FI-RSV stimulated the Th2 type allergic response [23–25, 29–32]. In contrast, animals immunized and challenged with wt RSV developed Th1 type antibody protection against RSV. Likewise, natural RSV infection produces a Th1 mediated immune response against RSV. However, the most desirable immunity against any kind of pathogen is a balanced Th1/Th2 response. Even though the exact mechanism of FI-RSV mediated enhanced disease was not fully understood, Murphy et al. suggested that formalin treatment altered the protective epitopes of F and G proteins and failed to produce neutralizing antibodies against real RSV infections. They also reported that the sera from FI-RSV immunized recipients did not neutralize RSV* in vitro* due to the lack of RSV specific neutralizing antibodies compared to the sera from wt RSV immunized recipients [23]. Consequently, the native form of RSV F is required to produce neutralizing antibodies and provide immunization against RSV infections. DNA vaccines are thought to be more advantageous due to the processing of antigens in their native forms by eukaryotic cells and due to the efficient presentation of antigens to antigen presenting cells. Thus, antibodies produced against the recombinant antigen expressed in the target host would easily recognize native nondenatured proteins of the pathogen and provide more efficient and specific protection against real pathogens compared to the recombinant protein vaccines expressed in bacteria [33]. In a previous study, we developed a DNA vaccine containing a region of RSV F (412–524 amino acids) conjugated with a modified cholera toxin gene and used to immunize mice which resulted in higher immune response [24].

As mentioned in the FI-RSV vaccine trial, the native form of the RSV F protein is very crucial in stimulating the protective immune response against RSV. Major structural changes in the RSV F protein may lead to disease exacerbation and allergic outcomes. The RSV F DNA vaccine is a preferred immunogen compared to the recombinant RSV F protein produced* ex vivo*. For DNA vaccinations, the intramuscular injection route is the best route that ensures antigen expression and native conformation. Besides the native structure, another advantage of DNA vaccination is that it elicits the Th1 biased immune response due to its endogenous expression and presentation to the immune cells, which is a favorable response for protection from pathogens [13, 34]. The Th1 immune system and the production of neutralizing antibodies are very important for protection from reinfection, which confers long term immunity by recruiting memory B cells. When the host encounters the same pathogen again, memory B cells abruptly produce pathogen specific neutralizing antibodies and immediately inactivate the pathogen before it enters into the host and starts infection [33]. We tested the ability of serum collected from RSV F immunized mice to neutralize RSV* in vitro*. Previous studies have shown that serum from FI-RSV infected mice does not neutralize RSV infection due to the altered structure of the RSV F protein [23].

Based on previous studies, distinct administration routes of the DNA vaccine evoke different types of immune responses. Our study was designed based on the previous DNA vaccine study where intramuscular injection of a DNA vaccine stimulated a moderate T cell response and antibody production compared to the oral administration, which induced a strong T cell response and weak antibody response [35].

RSV vaccine development has been hampered by the failure of previous vaccine trials that led to death of children. The main immunological event responsible of failure of the vaccine was induction of a predominant Th2 response that enhanced RSV disease following natural infection. Our study aimed at developing a safe DNA vaccine that induced a Th1 mediated antibody response. This study provides a basis for future RSV vaccine development that could benefit from DNA vaccine designs and may consider combination of DNA vaccine immunizations followed by traditional recombinant vaccine immunizations for higher protection from RSV infections.

## Figures and Tables

**Table 1:** The sequences of PCR/qPCR primers and probes.

Names of the primers	Sequences of the primers
RSV F forward primer	GGATCCACCATGATGGTCCTCAAAGCAAATGCAATTACCAC
RSV F reverse primer	CCACCGCGGCCGCTTATCATTGTCGACCAATATTATTTATACCACTC
GFP forward primer	GGATCCACCATGGTGAGCAAGGGCGAGGAGCTGTTCACCGG
GFP reverse primer	CCACGCGGCCGCTCATTACTTGTACAGCTCGTCCATGCCGTGAGTGATCC
RSV F QPCR forward primer	AACAGATGTAAGCAGCTCCGTTATC^*∗*^
RSV F QPCR reverse primer	CGATTTTTATTGGATGCTGTACATTT^*∗*^
RSV F QPCR probe	TGCCATAGCATGACACAATGGCTCCT^*∗*^

^*∗*^According to the sequences published by Mentel et al. [27]. All sequences are given 5′-3′ direction.

**Table 2:** Th2/Th1 (IgG1/IgG2a < 1 and IgG2b/IgG2a < 1) antibody ratios. IgG isotypes were detected by ELISA from serum samples of BALB/c mice (RSV-infected and PF DNA-immunized) on day 49. Data is presented as an average of triplicates performed twice.

	IgG1/IgG2a		IgG2b/IgG2a	
	RSV	PF DNA	RSV	PF DNA
Day 14	0.168	0.679	0.911	0.850
Day 28	0.553	0.443	0.827	0.666
Day 49	0.392	0.236	0.767	0.819
